# Angiotensin-converting enzyme gene insertion/deletion polymorphism and susceptibility to psoriasis: a systematic review and meta-analysis

**DOI:** 10.1186/s12881-019-0943-3

**Published:** 2020-01-08

**Authors:** Mazaher Ramezani, Elisa Zavattaro, Masoud Sadeghi

**Affiliations:** 1 Molecular Pathology Research Center, Imam Reza Hospital, Kermanshah University of Medical Sciences, Kermanshah, 6714415153 Iran; 2 Dermatology Unit, Department of Translational Medicine, University of Eastern Piedmont “Amedeo Avogadro”, 28100 Novara, Italy; 3 Medical Biology Research Center, Kermanshah University of Medical Sciences, Kermanshah, 6714415185 Iran; 4 Students Research Committee, Kermanshah University of Medical Sciences, Kermanshah, 6715847141 Iran

**Keywords:** Psoriasis, Psoriatic arthritis, Angiotensin-converting enzyme, Polymorphism, Meta-analysis

## Abstract

**Background:**

Psoriasis is a multifactorial disorder, impacted by both genetic and environmental factors. Herein, a meta-analysis assessed the association of angiotensin-converting enzyme gene insertion/deletion (*ACE* I/D) polymorphism and psoriasis susceptibility.

**Methods:**

A systematic search was used in databases of PubMed/Medline, Scopus, Web of Science, and Cochrane Library up to January 2019 without language restriction. A dichotomous analysis was carried out by RevMan 5.3 using crude odds ratio (OR) and 95% confidence interval (CI) to investigate the association between *ACE* I/D polymorphisms and the risk of psoriasis. A funnel plot analysis was used by CMA 2.0 to estimate a significant existence of publication bias.

**Results:**

Out of 61 studies retrieved from the databases, 16 studies were included in the meta-analysis. The pooled ORs for models of D vs. I, DD vs. II, ID vs. II, ID + DD vs. II, and DD vs. II + ID genotypes were 0.96 [95%CI: 0.82, 1.12; *P* = 0.58], 0.99 [95%CI, 0.73, 1.36; *P* = 0.96], 0.81 [95%CI, 0.72, 0.91; p: 0.0003], 0.91 [95%CI, 0.73, 1.13; *P* = 0.40], and 1.05 [95%CI, 0.85, 1.30; *P* = 0.68], respectively. A significant difference between ACE polymorphisms in patients with/without family history for the disease [OR = 1.44; 95%CI: 1.24, 1.67; P < 0.001] and also in patients mild/severe psoriasis [OR = 0.70; 95%CI: 0.55, 0.88; P = 0.002] was identified.

**Conclusion:**

The results of the meta-analysis showed that *ACE* I/D polymorphism may be associated with psoriasis susceptibility, while ID genotype seemed to have a protective role in Caucasian patients affected by psoriatic arthritis and in studies with hospital-based controls.

## Background

Psoriasis is a chronic inflammatory skin disease with unclear etiology that has been correlated with abnormal plasma lipid metabolism and oxidative stress [1], and with a high incidence of cardiovascular diseases [2] Psoriasis is impacting 2 to 3% of the general population [3]. It is a serious condition that may have negative effect on quality of life [4]. The differences in prevalence and incidence show that psoriasis is related to ethnic and geographic variations, being generally more prevalent in the cold northern regions than in the tropical area with a lower prevalence in China and Japan compared to Europe, and is virtually absent in natives of the Andean region of South America [5]. It is a multifactorial disorder, impacted by both genetic and environmental factors and its genetic basis has been established among studies in twins and familial clustering [6]. Angiotensin-converting enzyme (ACE) is a zinc metallopeptidase encoded on chromosome 17q23 [7]. *ACE* polymorphisms include an insertion (I)/deletion (D) within the intron 16 able to incorporate the most genetic variables responsible for the variability of ACE activity in serum [8]. To date, the molecular mechanism of the association between *ACE* I/D polymorphism and psoriasis susceptibility has not fully elucidated [9]. Furthermore, it has been investigated that the use of ACE inhibitors can create or aggravate psoriasis in clinical practice [10]. Some studies suggest that *ACE* and its related products might have widespread effects on immune responses and skin inflammation [11]. In addition, three meta-analyses have been previously published on association of *ACE* polymorphism and psoriasis susceptibility [12–14], unless they did not pay attention to genotypes/allele distribution, quality assessment of the included studies, and did not analyze subgroups of patients according to different factors (i.e. psoriasis variants and ethnicity).

Therefore, the aim of the present meta-analysis was to assess genotypes and alleles distribution in psoriasis based on five genetic models and through the evaluation of studies quality and also considering the association between *ACE* I/D polymorphism and psoriasis susceptibility in case-control studies.

## Methods

The study was designed following the Preferred Reporting Items for Systematic Reviews and Meta-Analyses (PRISMA) guidelines [15].

### Identification of eligible studies

A systematic search in PubMed/Medline, Scopus, Web of Science, and Cochrane Library databases was conducted up to January 2019, without language restriction. The search terms or keywords were: “psoriasis”, “psoriatic” and “ACE”, “angiotensin-converting enzyme” and “polymorphism (s)”, “variant (s)”, “gene (s)”. One author (M.S) searched the databases for articles, checked the titles and abstracts of each article, and excluded the not relevant studies. Two authors (M.R and E.Z) reviewed the full-texts to select the studies that met the eligibility criteria. Inclusion criteria were represented by: (1) human case-control study; (2) any subtype of psoriasis (i.e. psoriasis vulgaris, psoriatic arthritis); (3) reporting *ACE* I/D polymorphism in psoriatic patients and controls; and (4) having sufficient data for calculating odds ratio (OR) and 95% confidence interval (95%CI). Exclusion criteria consisted of: (1) animal study; (2) review; (3) meta-analysis; and (4) case report and case series.

### Data extraction

The data for each study were extracted by one author (M.S) and consisted of the first author, publication year, genotype frequencies in patients and controls, source of controls (i.e. hospital-based, population-based), psoriasis subtype, genotyping method, *p*-value for the Hardy–Weinberg Equilibrium (HWE) for controls, ethnicity, gender, family history for psoriasis, age at onset, subtypes of psoriasis, and quality score. Another author (M.R) rechecked the reached data.

### Quality assessment

One author (M.R) assessed the quality of each retrieved article using the Newcastle-Ottawa Quality Assessment Scale questionnaire with a maximum total score of 9 for case-control study [16].

### Statistical analysis

A dichotomous analysis was carried out by Review Manager 5.3 (RevMan 5.3) using crude OR and 95% CI to indicate the association between *ACE* I/D polymorphisms and psoriasis susceptibility. The association was assessed using five genetic models (allelic, heterozygote, homozygote, dominant, and recessive models) [17]. In addition, within- and between-study variations and heterogeneities were evaluated using Cochran’s Q-statistic: such test considers the null hypothesis in which all studies assessed the same effect (significance level: *P* < 0.05). The effect of heterogeneity was quantified using I^2^ statistic to measure the degree of inconsistency across studies, with a range between 0 and 100% that represents the proportion of between-study variability attributable to heterogeneity rather than chance [18]. A statistically significant heterogeneity was obtained with *P*-value < 0.1 (I^2^ > 50%). In case no significant heterogeneity was obtained, fixed-effect model was applied in order to estimate the pooled ORs and CI values. Otherwise, we applied the random-effect model [19]. Chi-square test was used to calculate the HWE in the control groups whether observed genotype frequencies in controls conformed to HWE expectations.

Subgroup analysis was managed according to ethnicity, psoriasis subtype, source of controls and normal HWE. In addition, distributions of alleles and genotypes of *ACE* I/D polymorphism were calculated by IBM SPSS version 22 using binary logistic regression based on some characteristics of psoriatic patients. A funnel plot analysis was used by the Comprehensive Meta-Analysis software version 2.0 (CMA 2.0) using both Egger’s and Begg’s tests with *P*-value (two-tailed) < 0.05 was estimated as significant existence of publication bias. To evaluate the consistency or stability of the results, the sensitivity analysis was used by removing one study, cumulative analysis, and excluding the studies without HWE in controls.

## Results

**Fig. 1:**
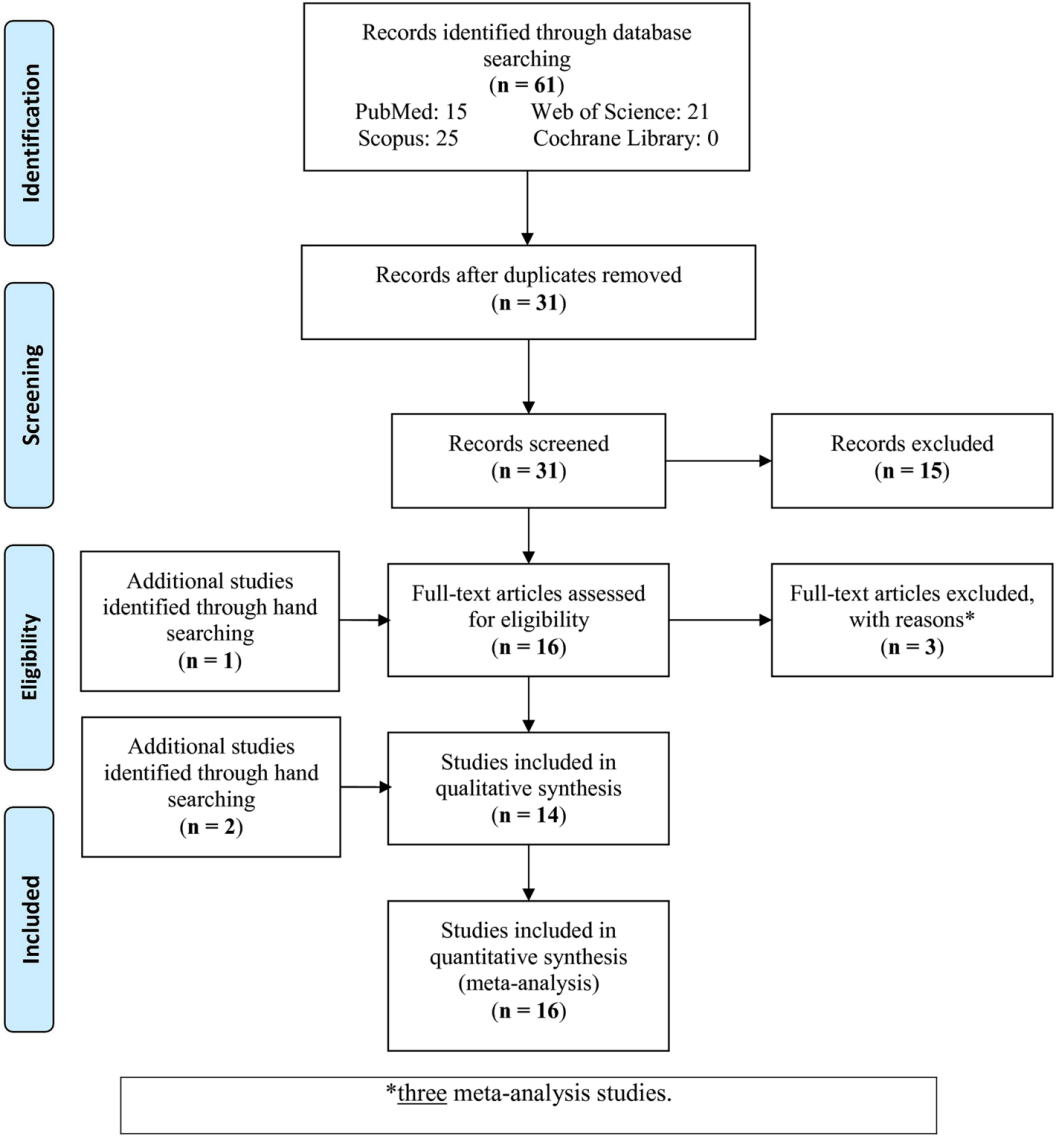
Flow-chart of the study

The schematic representation of the study selection process is shown in Fig. 1. Out of 61 studies retrieved from the databases after excluding duplicate and not relevant studies, the full-texts of 16 studies and another study identified by hand searching were assessed for eligibility (a total of 17 full-texts). After checking the full-texts, three studies were recognized as meta-analyses and consequently they were excluded. But checking the references of previous meta-analyses related to the subject, two other studies [20, 21] were added that we didn’t find their full-text; since they had previously included in other meta-analyses, and since all the required data were extracted from other meta-analyses. In conclusion, 16 studies were included in the meta-analysis.

### Study characteristics

Some important characteristics of the studies involved in the present meta-analysis are shown in Table 1. The studies were published from 1999 to 2018. Twelve studies reported their research in Caucasian ethnicity [21–23, 25–30, 32–34] and four studies [9, 20, 24, 31] in Asian ethnicity. Among Caucasian ethnicity, four studies [23, 26, 27, 33] were conducted in Arab population, four studies [21, 25, 28, 29] enrolled European population, while the remaining four studies [22, 30, 32, 34] evaluated other populations (Pakistani, Iranian and Turkish populations). The meta-analysis included 3003 psoriatic patients and 3689 controls. With regard to the controls source, nine studies [9, 20, 23–27, 31, 34] enrolled hospital-based patients, two studies [29, 30] were population-based, and five studies [21, 22, 28, 32, 33] enrolled controls of unknown origin. In most of the considered studies, the ratio of patients affected by different psoriasis subtypes was not reported [9, 20, 21, 24, 31, 32, 34], while only 5 and 4 studies were conducted in groups of patients affected predominantly by psoriasis vulgaris [22, 25, 26, 28, 33] and psoriatic arthritis [23, 27, 29, 30], respectively. In all studies the used genotyping method was polymerase chain reaction (PCR). In two out of 16 studies, the genotype frequencies of controls [27, 28] didn’t follow HWE.

**Table 1:** Characteristics of the studies included in the meta-analysis (*n* = 16)

Genotyping												
Study, year	Ethnicity	Psoriasis			Control			Source of controls	Subtype of psoriasis	Genotyping method	P-value for HWE for controls	Score^a^
		II	ID	DD	II	ID	DD					
Vasku, 1999 [21]	Caucasian (European)	40	111	49	45	104	59	Unknown	Unknown	PCR	0.947	NA
Ozkur, 2004 [22]	Caucasian (Turkish)	12	40	34	28	69	57	Unknown	Vulgaris (94.2%)	PCR	0.378	7
Al-Awadhi, 2007 [23]	Caucasian (Arab)	7	19	25	14	45	41	Hospital- based	Arthritis	PCR	0.770	8
Chang, 2007 [24]	Asian	172	108	32	287	265	63	Hospital- based	Unknown	PCR	0.873	7
Liu, 2007 [20]	Asian	31	38	19	23	48	24	Hospital- based	Unknown	PCR	0.917	NA
Weger, 2007 [25]	Caucasian (European)	61	92	54	35	93	54	Hospital- based	Vulgaris	PCR	0.653	7
Nagui, 2012 [26]	Caucasian (Arab)	9	13	8	6	8	6	Hospital- based	Vulgaris	PCR	0.371	8
Shehab, 2008 [27]	Caucasian (Arab)	2	2	9	19	18	74	Hospital- based	Arthritis	PCR	**< 0.001**	6
Veletza, 2008 [28]	Caucasian (European)	2	11	14	5	7	15	Unknown	Vulgaris	PCR	**0.038**	7
Coto-Segura, 2009 [29]	Caucasian (European)	38	124	106	34	145	93	Population- based	Arthritis	PCR	0.050	6
Yang, 2014 [9]	Asian	350	269	49	304	299	65	Hospital- based	Unknown	PCR-RFLP	0.491	7
Munir, 2016 [30]	Caucasian (Pakistani)	118	239	129	88	299	184	Population- based	Arthritis (92%)	PCR	0.063	6
Huang, 2017 [31]	Asian	55	71	35	119	111	26	Hospital- based	Unknown	PCR	0.987	6
Agha, 2018 [32]	Caucasian (Pakistani)	72	87	74	90	122	51	Unknown	Unknown	PCR	0.405	6
Elneam, 2018 [33]	Caucasian (Arab)	18	33	22	22	17	8	Unknown	Vulgaris	PCR	0.157	7
Tanhapour, 2018 [34]	Caucasian (Iranian)	16	57	27	8	50	42	Hospital- based	Unknown	PCR-RFLP	0.191	6

***Abbreviations****: PCR Polymerase chain reaction; RFLP restriction fragment length polymorphism.* ^*a*^*Based on* the Newcastle-Ottawa Quality Assessment Scale

**Fig. 2:**
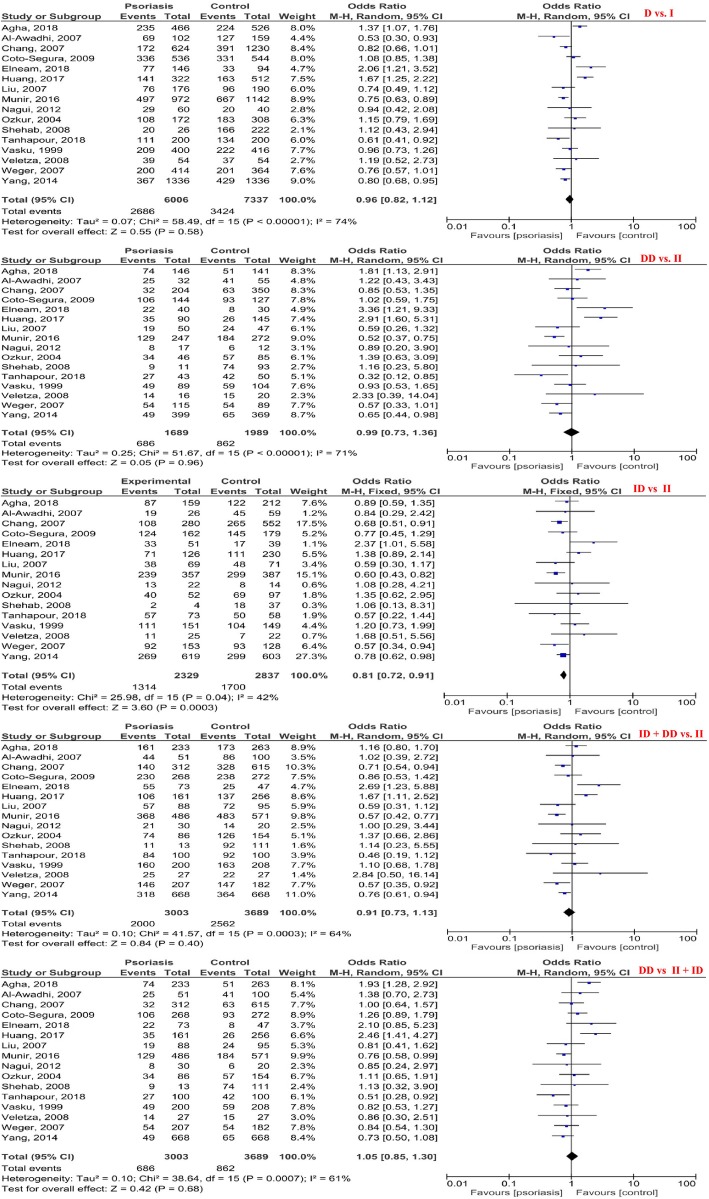
Forest plot of psoriasis susceptibility related to angiotensin-converting enzyme insertion/deletion (I/D) polymorphism based on five genetic models: (**a**) D vs. I, (**b**) DD vs. II, (**c**) ID vs. II, (**d**) ID + DD vs. II, and (**e**) DD vs. II + ID

Forest plot of the psoriasis susceptibility related to *ACE* I/D polymorphisms based on five genetic models is identified in Fig. 2. The pooled ORs of D versus I, DD versus II, ID versus II, ID + DD versus II, and DD versus II + ID models were 0.96 [95%CI: 0.82, 1.12; *P* = 0.58; I^2^ = 74% (P_heterogeneity_ (P_h_) < 0.00001)], 0.99 [95%CI: 0.73, 1.36; *P* = 0.96; I^2^ = 71% (P_h_ < 0.0001)], 0.81 [95%CI: 0.72, 0.91; *P* = 0.0003; I^2^ = 42% (P_h_ = 0.04)], 0.91 [95%CI: 0.73, 1.13; *P* = 0.40; I^2^ = 64% (P_h_ = 0.0003)], and 1.05 [95%CI: 0.85, 1.30; *P* = 0.68; I^2^ = 61% (P_h_ = 0.0007)], respectively. Therefore, the presence of ID genotype had a significant slight protective effect against psoriasis development.

### Subgroup analysis

The results of psoriasis susceptibility related to ACE I/D polymorphisms on the basis of different considered variables are shown in Table 2. With regard to ethnicity, the analyses showed that there was no risk of psoriasis related to *ACE* I/D polymorphisms in East Asian populations, but the presence of ID genotype had a slight protective effect against the disease [OR = 0.82; 95%CI: 0.69, 0.97]. Based on three models of DD versus II, ID versus II, and ID + DD versus II the pooled OR was 0.69 (95%CI: 0.52, 0.91), 0.66 (95%CI: 0.50, 0.85), and 0.67 (95%CI: 0.52, 0.86), respectively. Such results showed that DD and ID genotypes had protective roles in psoriatic arthritis, unless this was not valid for psoriasis vulgaris type. The ID genotype had a significantly decreased susceptibility to psoriasis in studies conducted in hospital-based populations based on heterozygote model (OR = 0.77, 95%CI: 0.67, 0.90). Caucasian population was evaluated in three groups, Arab, European and the other group. The other group consisted of Iran, Pakistan and Turkey. With regard to the Caucasian population, the ID genotype had a significant protective role in psoriasis if compared with other genotypes in non-Arab and non-European population (the other group) (OR = 0.73, 95%CI: 0.58, 0.92).

**Table 2:** Analysis of psoriasis susceptibility related to angiotensin-converting enzyme insertion/deletion (I/D) polymorphism based on the studies with normal Hardy–Weinberg Equilibrium when considering controls**,** ethnicity, psoriasis subtype, controls source, Caucasian population

Variable (no. of study)	D vs. I	DD vs. II	ID vs. II	ID + DD vs. II	DD vs. II + ID
	OR (95%CI), I^2^ (%), P_h_	OR (95%CI), I^2^ (%), P_h_	OR (95%CI), I^2^ (%), P_h_	OR (95%CI), I^2^ (%), P_h_	OR (95%CI), I^2^ (%), P_h_
Overall (16)	0.96 (0.82, 1.12), 74, < 0.00001	0.99 (0.73, 1.36), 71, < 0.00001	**0.81 (0.72, 0.91), 42, 0.04**	0.91 (0.73, 1.13), 64, 0.0003	1.05 (0.85, 1.30), 61, 0.0007
Ethnicity					
East Asian (4)	0.95 (0.68, 1.32),86, < 0.0001	0.99 (0.51, 1.93), 84, 0.0004	0.82 (0.61, 1.10), 63, 0.05	0.86 (0.59, 1.25), 79, 0.003	1.09 (0.65, 1.84), 77, 0.005
Caucasian (12)	0.96 (0.79, 1.17), 70, 0.0001	0.99 (0.68, 1.45), 67, 0.0005	**0.82 (0.69, 0.97), 39, 0.08**	0.95 (0.71, 1.27), 59, 0.004	1.03 (0.81, 1.32), 57, 0.007
Psoriasis subtype					
Arthritis (4)	0.83 (0.61, 1.12), 66, 0.03	**0.69 (0.52, 0.91), 49, 0.12**	**0.66 (0.50, 0.85), 0, 0.78**	**0.67 (0.52, 0.86), 8, 0.35**	1.04 (0.73, 1.48), 53, 0.09
Vulgaris (5)	1.12 (0.77, 1.65), 65, 0.02	1.29 (0.62, 2.68), 63, 0.03	1.19 (0.65, 2.21), 60, 0.04	1.29 (0.64, 2.63), 71, 0.009	1.02 (0.76, 1.37), 0, 0.48
Source of controls					
Hospital-based (9)	0.84 (0.68, 1.04),72, 0.0004	0.84 (0.55, 1.29), 68, 0.002	**0.77 (0.67, 0.90), 23, 0.24**	0.80 (0.61, 1.04), 56, 0.02	0.97 (0.71, 1.32), 58, 0.01
Others (7)	1.12 (0.88, 1.42), 77, 0.0003	1.22 (0.74, 2.01), 77, 0.0002	1.00 (0.72, 1.40), 59, 0.02	1.11 (0.75, 1.64), 72, 0.001	1.14 (0.83, 1.56), 68, 0.005
Caucasian population					
Arab (4)	1.04 (0.52, 2.06), 75, 0.008	1.65 (0.91, 3.00), 2, 0.38	1.43 (0.81, 2.53), 0, 0.47	1.56 (0.93, 2.62), 7, 0.36	1.41 (0.89, 2.23), 0, 0.68
Europe (4)	0.94 (0.81, 1.10), 21, 0.28	0.85 (0.62, 1.16), 18, 0.30	0.84 (0.63, 1.12), 47, 0.13	0.84 (0.64, 1.10), 48, 0.13	0.99 (0.79, 1.24), 4, 0.37
Other (Iran, Pakistan, and Turkey) (4)	0.93 (0.64, 1.34), 85, 0.0001	0.83 (0.38, 1.84), 86, < 0.0001	**0.73 (0.58, 0.92), 41, 0.17**	0.82 (0.49, 1.36), 75, 0.007	0.97 (0.57, 1.66), 84, 0.0003

*Bold number means significant (*P* < 0.05). P_h_ equals to P_heterogeneity_

### Sensitivity analysis

Out of 16 studies included in the meta-analysis, two studies [27, 28] were excluded since *P*-value of HWE for controls was less than 0.05 (Table 3). Notwithstanding, the new analysis showed that the results were unchanged with just a decreased susceptibility to psoriasis among patients carrying ID genotype (OR = 0.80, 95%CI: 0.71, 0.90). In addition, other analyses - one study removed and cumulative analysis- didn’t change the result of previous overall analysis and therefore they showed the stability of the previous overall result.

**Table 3:** Analysis of psoriasis susceptibility related to angiotensin-converting enzyme insertion/deletion (I/D) polymorphism after excluding the studies without normal Hardy–Weinberg Equilibrium

Variable (no. of study)	D vs. I	DD vs. II	ID vs. II	ID + DD vs. II	DD vs. II + ID
	OR (95%CI), I^2^ (%), P_h_	OR (95%CI), I^2^ (%), P_h_	OR (95%CI), I^2^ (%), P_h_	OR (95%CI), I^2^ (%), P_h_	OR (95%CI), I^2^ (%), P_h_
Normal HWE (14)	0.95 (0.80, 1.12), 78, < 0.00001	0.97 (0.70, 1.34), 74, < 0.00001	**0.80 (0.71, 0.90), 47, 0.03**	0.89 (0.72, 1.11), 67, 0.0002	1.05 (0.84, 1.32), 66, 0.0002

*Bold number means significant (*P* < 0.05). P_h_ equals to P_heterogeneity_

### Genotype distribution

The alleles and genotypes distribution of *ACE* I/D polymorphism on the basis of the differences in patient’s characteristics are shown in Table 4. In detail, only three studies [20–22] considered gender, six studies [9, 20, 22, 23, 25, 28] reported family history for psoriasis, nine studies [9, 22–25, 27–29, 33] considered the age at the onset, one study [6] reported type of psoriasis, and three studies [9, 28, 31] reported severity of the disease. The results showed significant difference between ACE polymorphisms in patients with family history (familial) versus those without family history (sporadic) for the disease [OR = 1.44; 95%CI: 1.24, 1.67; P < 0.001]. When considering psoriasis severity (grouped among severe or mild disease), a significant difference was obtained [OR = 0.70; 95%CI: 0.55, 0.88; P = 0.002]. Therefore, the II genotype was significantly more represented in familial patients than in sporadic patients and the DD genotype was more frequent in severe than in mild psoriasis. There was no significant difference in terms of gender, age at the onset, and type of psoriasis among groups of patients.

**Table 4:** Distribution of alleles and genotypes of angiotensin-converting enzyme insertion/deletion (I/D) polymorphism with respect to patient characteristics in psoriasis

Variable (no. of study)	II	ID	DD	I	D	OR (95%CI), *P*-value
Sex (3)						
Male (*n* = 194) vs. Female (*n* = 180)	41 (21%) vs. 42 (23%)	102 (53%) vs. 87 (49%)	51 (26%) vs. 51 (28%)	184 (47.4%) vs. 171 (47.5%)	204 (52.6%) vs. 189 (52.5%)	0.99 (0.74, 1.33), 0.983
Family history (6)						
Positive (*n* = 506) vs. Negative (*n* = 685)	224 (44%) vs. 223 (32%)	194 (38%) vs. 308 (45%)	88 (18%) vs. 154 (22%)	642 (63.4%) vs. 754 (55%)	370 (36.6%) vs. 616 (45%)	**1.44 (1.24, 1.67), < 0.001**
Age at onset (9)						
Early-onset (*n* = 1522) vs. Late-onset (*n* = 715)	516 (33.9%) vs. 265 (37.1%)	666 (43.8%) vs. 298 (41.7%)	340 (22.3%) vs. 152 (21.2%)	1698 (55.8%) vs. 828 (57.9%)	1346 (44.2%) vs. 602 (42.1%)	0.93 (0.82, 1.04), 0.208
Type of psoriasis (2)						
Type Ӏ (*n* = 256) vs. Type ӀӀ (*n* = 204)	151 (59%) vs. 109 (53.4%)	90 (35.2%) vs. 71 (34.8%)	15 (5.8%) vs. 24 (11.8%)	392 (76.6%) vs. 289 (71%)	120 (23.4%) vs. 119 (39%)	1.31 (0.99, 1.74), 0.060
Severity (3)						
Mild (*n* = 807) vs. Severe (*n* = 202)	332 (41.1%) vs. 74 (36.6%)	355 (44%) vs. 71 (35.1%)	120 (14.9%) vs. 57 (28.2%)	1019 (63.1%) vs. 219 (54.2%)	595 (36.9%) vs. 185 (45.8%)	**0.70 (0.55, 0.88), 0.002**

*Bold number means significant (*P* < 0.05); vs. = versus; Early-onset = age at onset ≤40 years; Late-onset = age at onset > 40 years; Type Ӏ = having a positive family history and early-onset disease; Type ӀӀ = having a negative family history and late-onset disease; Mild severity = PASI < 10; Severe psoriasis = PASI ≥10 [PASI = Psoriasis Area and Severity Index] [35]

### Quality assessment

The evaluation of quality for each study is shown in Table 1. Unfortunately, the full-text of two studies [20, 21] was not available for the quality assessment. In detail, eight studies had high quality (score ≥ 7).

### Publication bias

**Fig. 3:**
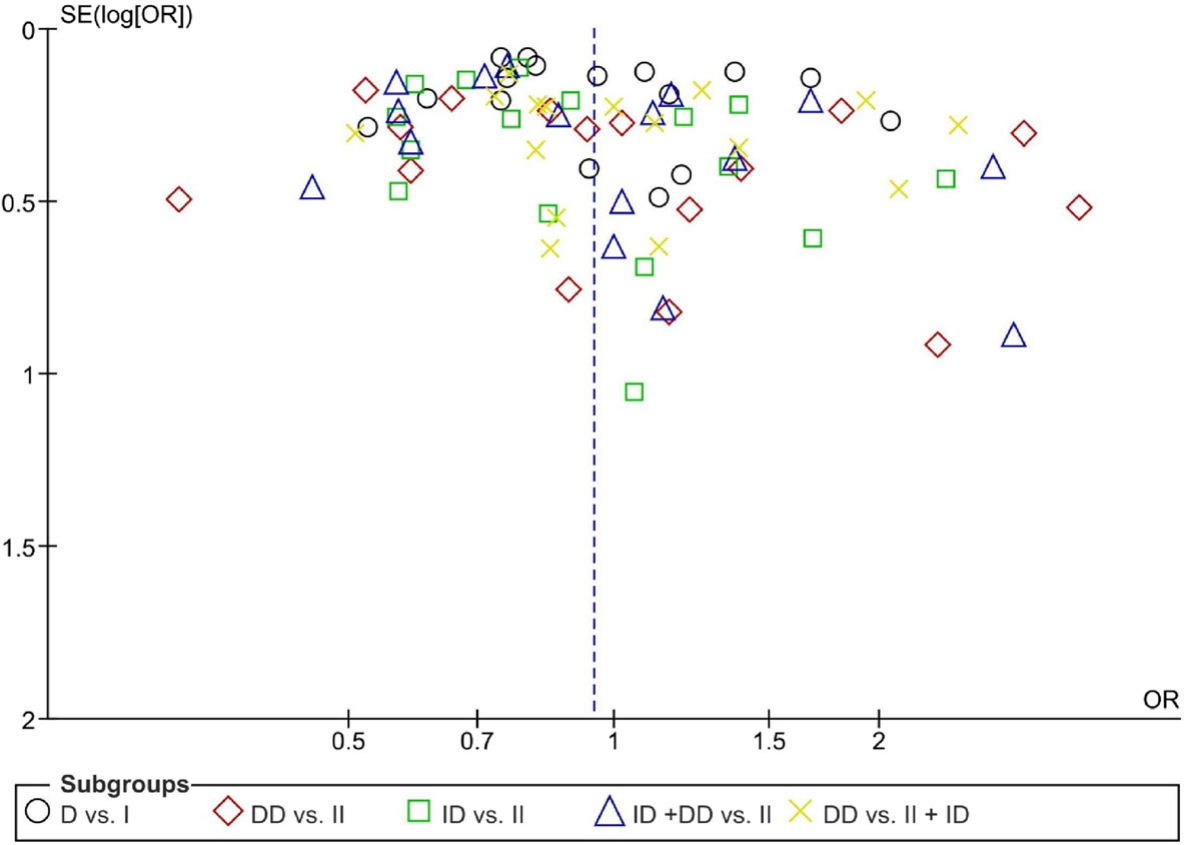
Funnel plot of the risk of psoriasis related to angiotensin-converting enzyme insertion/deletion (I/D) polymorphism based on five genetic models

We checked publication bias for overall analysis using both Egger’s and Begg’s tests (Fig. 3). The results showed that both tests didn’t reveal the existence of publication bias between the studies in each model analyses (*P* > 0.05).

## Discussion

The present meta-analysis investigated the association between *ACE* I/D polymorphisms with psoriasis susceptibility and also the distribution of genotypes in psoriatic patients. The results indicated the ID genotype is significantly associated with decreased risk of psoriasis development. In addition, in a further subgroup analysis, such genotype resulted to be protective against psoriasis and psoriatic arthritis in Caucasian patients versus non-Arab and non-European population. The same genotype showed to be less represented among patients in hospital-based studies. Out of all studies included in the meta-analysis, four studies [9, 23, 30, 34] showed a significant decreased psoriasis susceptibility, while three studies [31–33] reported a significant increased risk to develop psoriasis in subjects carrying the D allele. Similarly, the DD genotype was associated to a significant decreased psoriasis susceptibility in four studies [9, 25, 30, 34] and increased risk three studies [31–33], respectively. Moreover, the ID genotype showed a significant decreased risk of psoriasis in four studies [9, 24, 25, 30] and a significant elevated susceptibility to the disease in one study [33].

Previously, a meta-analysis conducted in ten studies and checking for the association between *ACE* I/D polymorphisms and psoriasis susceptibility [12] suggested that the ID genotype was a predisposing factor for psoriasis in East Asian subjects. A further meta-analysis evaluated eight studies [13] showed that in Asian ethnicity, the II genotype and I allele were associated with increased susceptibility to psoriasis, whereas the ID genotype seemed to have a protective role. In addition, a meta-analysis enrolling only five studies [14] concluded that only the DD + ID genotype showed significant association with psoriasis (OR = 0.75; *P* = 0.006).

The present meta-analysis included 16 studies, and described the different genotypes and alleles’ distribution in the groups and subgroups analysis had also been performed. Furthermore, a careful quality assessment of the involved studies had been conducted, in order to consider only the high-quality studies and to provide a further strength to our results.

A possible explanation for its role in psoriasis may be related to the fact that the *ACE* II genotype reduces ACE activity in skin and may prolong or augment activation of the kallikrein–kinin system, thereby increasing the risk for psoriasis [24]. The activation of the kallikrein–kinin system in plasma and tissue has also been associated with psoriasis [36, 37]. Several studies indicated that ACE is a major and effective factor in creating angiotensin II (Ang II) and inactivating bradykinin [11, 38]. Plasma and tissue ACE levels have been found to be related to the D allele of the *ACE* I/D polymorphism, with DD genotypes having the highest and II genotypes having the lowest ACE activity [39]. Increased levels of serum ACE, IL-6 and IL-8 in psoriasis patients were due to the important role of ACE in inflammation. ACE converts Ang I into Ang II and inactivates bradykinin [40], moreover Ang II activates cytokines like IL-6 and IL-8, thus exerting proinflammatory effects [11]. This shows an important role of ACE in the pathogenesis of psoriasis.

In the previously published data, one study [30] reported the ӀӀ genotype and I allele frequencies were significantly higher in male patients affected by psoriasis, whereas no association was observed in female patients. It might be supposed that such gender-based discrepancies may be due to differences in the renin-angiotensin system among men and women and the mechanism might involve the role of sex hormones. Another study [22] didn’t find any difference between gender and genotype frequencies of *ACE* I/D polymorphism. In addition, no significant difference was found between polymorphisms and age at onset [9, 22–26, 29], type of psoriasis [9, 22, 30], disease severity [9, 29], and family history [23, 29]. In contrast, the II genotype and I allele frequencies in patients with familial history of psoriasis and type I psoriasis were higher than patients with sporadic psoriasis and type II [22]. Another study [26] confirmed this result in familial psoriasis. Elneam et al. [33] showed that the DD genotype was more common in case of severe psoriasis vulgaris and the ID was more frequent in non-severe psoriasis vulgaris patients. The present meta-analysis failed to identify a significant difference between gender, age at the onset, and type of psoriasis with genotype frequencies, but the II genotype frequency was significantly higher in patients with positive family history for psoriasis than in sporadic patients; moreover the DD genotype was significantly more represented in subjects with severe than in those with non-severe disease.

The differences between our results and those with other previous studies may be due to diverse factors, thus including racial/geographical difference, number of male/female patients in the considered study and also to the genetic heterogeneity and multifactorial etiology of psoriasis [30]. Also, Ethnic factors and differences among genotyping assay techniques might contribute to the variability between reports evaluating the role of the *ACE* I/D polymorphisms [41]. In our meta-analysis, we have detected that ethnicity, psoriasis subtype, and source of controls can represent significant factors in terms of susceptibility to develop such disease.

Our study presents several important limitations: i) a high heterogeneity among the considered studies was identified; ii) the number of ethnic groups in the studies was limited; iii) in many studies the psoriasis subtypes and source of controls were not clearly specified. Notwithstanding, despite these limitations, there was no publication bias in the analyses.

## Conclusions

Summing up, the results of the present meta-analysis showed that *ACE* I/D polymorphisms may be associated with psoriasis susceptibility and, in detail the ID genotype seemed to have a protective role, mainly in Caucasian patients, against psoriatic arthritis, and in the studies considering hospital-based controls. In addition, the DD genotype showed a protective role against psoriatic arthritis. In conclusion, the distributions of genotypes of *ACE* I/D polymorphism were different when the patients were compared in the terms of family history and severity of the disease.

## Data Availability

The datasets used and/or analyzed during the current study available from the corresponding author on reasonable request.

## Abbreviations

ACE I/D: Angiotensin-converting enzyme gene insertion/deletion
CI: Confidence interval
HWE: Hardy–Weinberg Equilibrium
OR: Odds ratio
PRISMA: The Preferred Reporting Items for Systematic Reviews and Meta-Analyses
